# λ-Carrageenan improves the antitumor effect of dendritic cellbased vaccine

**DOI:** 10.18632/oncotarget.15610

**Published:** 2017-02-22

**Authors:** Jinyao Li, Adila Aipire, Jinyu Li, Hongge Zhu, Yanping Wang, Wenjia Guo, Xiaoqin Li, Jia Yang, Chunling Liu

**Affiliations:** ^1^ Affiliated Tumor Hospital of Xinjiang Medical University, Urumqi 830011, China; ^2^ College of Life Science and Technology, Xinjiang University, Urumqi 830046, China; ^3^ XinJiang DingJu Biotech CO., LTD, Urumqi 830000, China; ^4^ Bayin Guoleng Vocational and Technical College, Korla 841000, China

**Keywords:** λ-carrageenan, TLR4, dendritic cell-based vaccine, cancer immunotherapy, cellular responses

## Abstract

In this study, we investigated the effect of λ-carrageenan on the maturation and function of dendritic cells (DCs) and its adjuvant effect on DC-based vaccine. We found that λ-carrageenan dose-dependently decreased the endocytosis of DCs, promoted DC maturation and increased cytokine production through TLR4 mediated signaling pathway. λ-carrageenan treatment also enhanced the ability of DCs in the stimulating allogenic splenocyte proliferation. In TC-1 tumor mouse model, HPV peptides pulsed λ-carrageenan-DC (HPV-CGN-DC) significantly inhibited tumor growth compared with control group. The frequencies of CD4^+^ and CD8^+^ T cells in spleens of tumor mice and their activation status were significantly increased in HPV-CGN-DC group, but the frequencies of natural regulatory T cells and CD11b^+^Gr-1^+^ cells were significantly decreased. Further, HPV-CGN-DC induced strong CD8^+^ T cell responses, which are negatively correlated with tumor volumes. The results suggested that λ-carrageenan promoted DC maturation through TLR4 signaling pathway and could be used as the adjuvant in DC-based vaccines.

## INTRODUCTION

Dendritic cells (DCs), as professional antigen presenting cells (APCs), can capture, process and present antigens to naïve T cells to activate antigen-specific immune responses. The activation status of DCs including the expression of co-stimulatory molecules and MHC I/II, and the secretion of cytokines determined the differentiation of CD4^+^ helper T cell (Th) subsets and CD8^+^ T cell responses [1]. IL-12 secreted by DCs promotes the induction of CD4^+^ Th1 cells and cytotoxic T lymphocytes (CTL) to suppress tumor growth [2, 3]. Therefore, DCs as antigen carrier were considered promising vaccine platform against tumors. Clinical trials of DC-based vaccines carried out in tumor patients have been proved its safety and capacity in the induction of antigen-specific immune responses [4]. However, the clinical efficacy of DC-based vaccines remains to be improved.

Adjuvant can be used to enhance the immunogenicity of various kinds of vaccines through different mechanisms, such as depot formation, induction of cytokines and chemokines, increase of antigen uptake, improvement of APC maturation and antigen presentation [5, 6]. Due to the safety, only a few of adjuvants including aluminum salts, MF59, AS03, AF03 and AS04 have been approved for use in human vaccines in the US and/or Europe [6, 7]. Lots of efforts were focused on the development of safe and effective adjuvants.

Carrageenans (CGNs) are mucopolysaccharides with high molecular weight from marine red seaweeds, which contained β-1,4 and α-1,3 glycosidic linkages [8]. According to the number and the position of sulfated ester, CGNs are mainly classified into three groups including kappa (κ)-, iota (ι)-, and lambda (λ)-CGNs [9, 10]. CGN has been widely used as a food additive in various kinds of foods, such as infant formula, and its safety has been confirmed by a large body of animal subchronic and chronic toxicity studies [11]. Recently, accumulating evidence has been shown that CGN has antitumor and immunomodulatory activities through toll-like receptor (TLR) 4 signaling pathway [8, 12–17]. Several studies also reported that CGN has adjuvant effect on the enhancement of immune responses induced by peptide/protein-based vaccines [12, 18]. However, it is still elusive whether CGN can regulate the maturation and function of DCs, and as adjuvant enhance the antitumor effect of DC-based vaccine. Here, we found that CGN could enhance DC maturation and cytokine production through TLR4 signaling pathway. Moreover, CGN stimulated DCs pulsed with human papillomavirus (HPV) peptides induced strong HPV-specific CD8^+^ T cell responses and greatly inhibited tumor growth in TC-1 tumor mouse model.

## RESULTS

### λ-CGN enhances DC maturation and cytokine production

**Figure 1:**
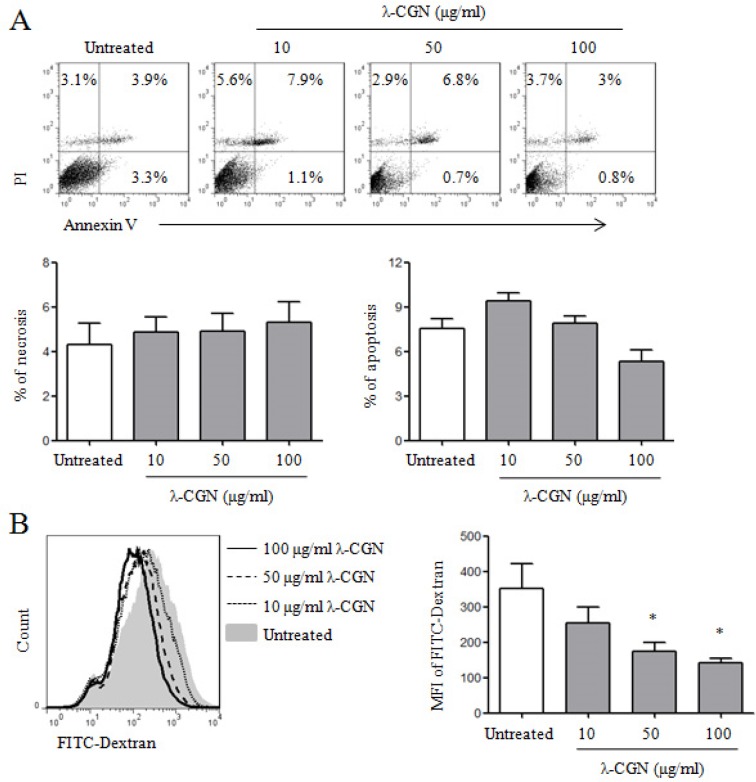
The effect of λ-CGN on DC viability and endocytosis On day 7, DCs were treated with 10, 50 and 100 μg/ml of λ-CGN for 12 h. **A**. After λ-CGN treatment, DCs were stained with Annexin V and PI and detected by flow cytometry. The representative dot plots are shown in upper panel. The summary of necrotic (Annexin V^−^PI^+^) and apoptotic (Annexin V^+^PI^−^ and Annexin V^+^PI^+^) cells is shown in lower panels. **B**. DCs treated with or without λ-CGN were inoculated with FITC-Dextran for 1 h. FITC-Dextran^+^ DCs were detected by flow cytometry (left panel). Summary of mean fluorescence intensity (MFI) is shown in right panel. Data are from 3 independent experiments. * *P*<0.05 (ANOVA) compared to untreated DCs.

It has been reported that λ-CGN has the immunomodulatory activity [8, 13–15]. Here, we investigated whether λ-CGN could regulate DC maturation and cytokine production. Firstly, we detected the effect of λ-CGN on the viability of DCs. Immature DCs were induced from bone marrow cells of C57BL/6 mice in the presence of GM-CSF and treated with 10, 50 and 100 μg/ml of λ-CGN. After 12 h, the viability of DCs was detected by Annexin V/PI staining. We found that λ-CGN did not increase the frequencies of necrosis (PI^+^Annexin V^−^) and apoptosis (PI^−^Annexin V^+^ and PI^+^Annexin V^+^) of DCs (Figure 1A), suggesting that it did not affect the viability of DCs in the selected concentrations. Next, we examined the endocytosis of DCs upon λ-CGN treatment. λ-CGN treated DCs were incubated with FITC-Dextran for 1 h and the mean fluorescence intensity (MFI) of FITC were analyzed by flow cytometry. Compared with untreated DCs, λ-CGN treatment dose-dependently decreased the endocytosis of DCs (Figure 1B).

**Figure 2:**
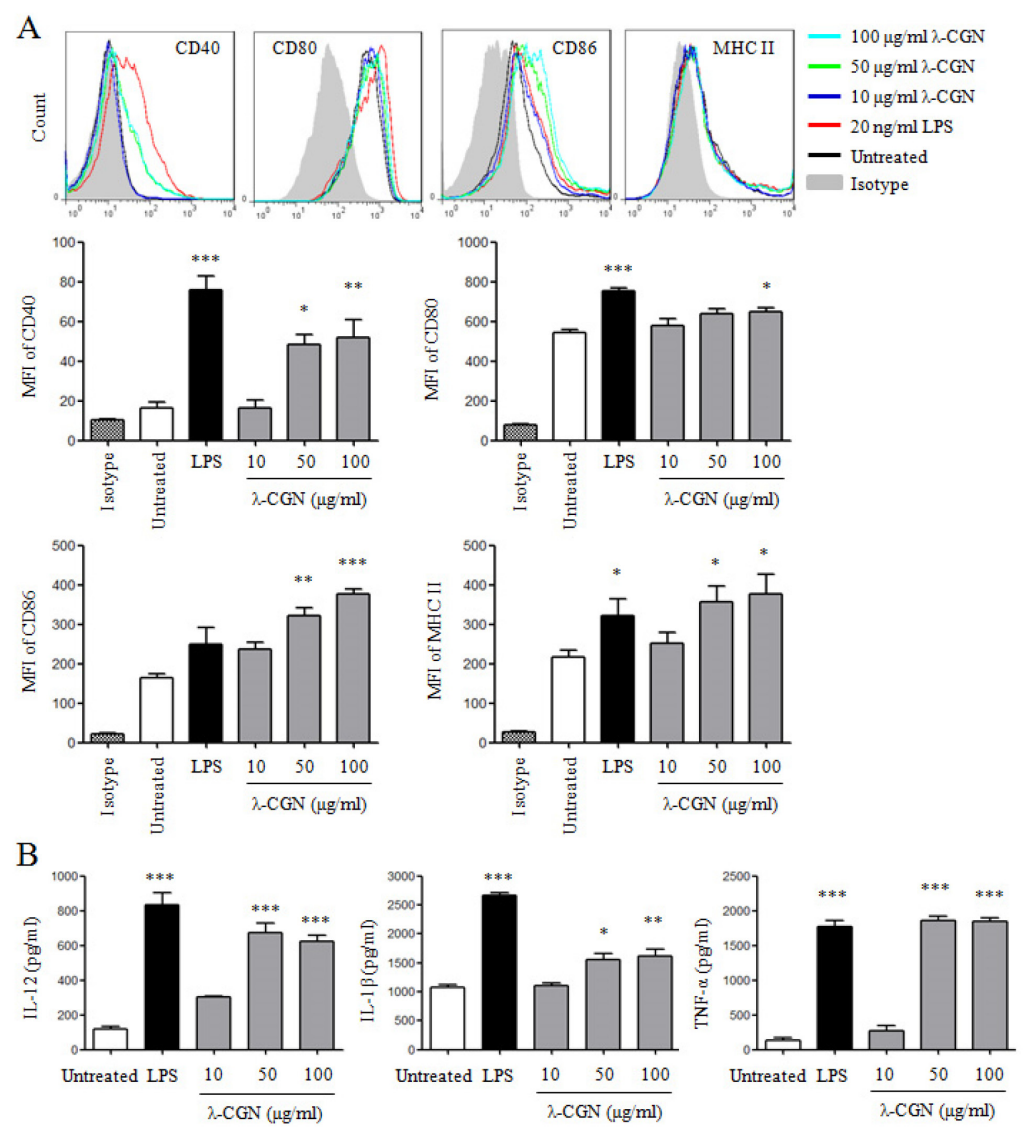
λ-CGN promotes DC maturation and cytokine production On day 7, DCs were treated with 10, 50 and 100 μg/ml of λ-CGN or 20 ng/ml of LPS for 12 h. **A**. The expressions of co-stimulatory molecules and MHC II on DCs were detected by flow cytometry (upper panels). Summary of MFI is shown in lower panels. **B**. The supernatants of the above samples were collected to detect the secretions of IL-12, IL-1β and TNF-α by ELISA. Data are from 4 independent experiments. * *P*<0.05, **P<0.01 and ***P<0.001 (ANOVA) compared to untreated DCs.

We further detected DC maturation and cytokine production after λ-CGN treatment. LPS was used as positive control. As shown in Figure 2A, λ-CGN treatment significantly up-regulated the expressions of CD40, CD80, CD86 and MHC II on DCs in a dose-dependent manner. The secretions of cytokines in supernatants of DCs were detected by ELISA. As shown in Figure 2B, λ-CGN treatment significantly increased the production of IL-12, IL-1β and TNF-α. These results suggested that λ-CGN promoted DC maturation and cytokine production.

### λ-CGN improves DC function

**Figure 3:**
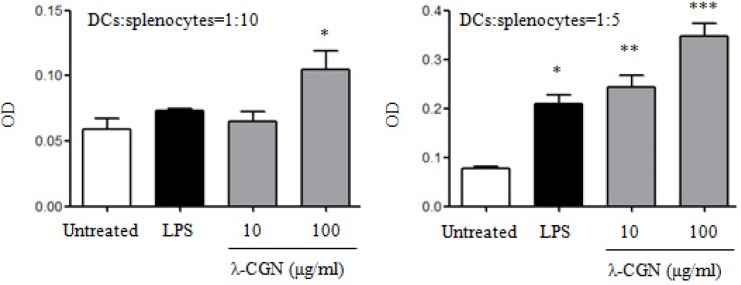
λ-CGN enhances DC function The function of LPS or λ-CGN treated DCs in the stimulation of lymphocyte proliferation were detected by MLR. DCs from C57BL/6 mice were treated with LPS or λ-CGN, and then co-cultured with splenocytes from BALB/c mice at 1:10 and 1:5 ratios. Data are from 2 independent experiments. * *P*<0.05, **P<0.01 and ***P<0.001 (ANOVA) compared to untreated DCs.

The function of λ-CGN treated DCs was determined by mixed lymphocyte reaction (MLR). λ-CGN treated DCs from C57BL/6 mice were co-cultured with splenocytes from BALB/c mice at the ratios of 1:10 and 1:5. Compared with untreated DCs, 100 μg/ml of λ-CGN treated DCs at the ratio of 1:10, and 10 and 100 μg/ml of λ-CGN treated DCs at the ratio of 1:5 significantly increased the proliferation of splenocytes (Figure 3), suggesting that λ-CGN improved DC function.

### TLR4 signaling pathway is involved in DC maturation induced by λ-CGN

**Figure 4:**
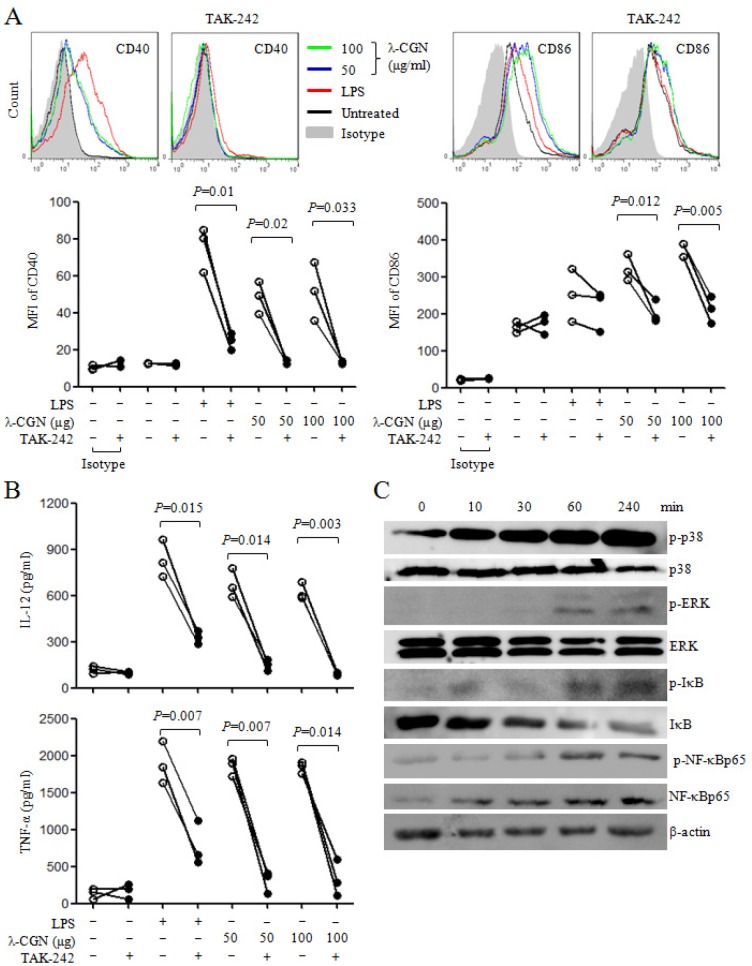
λ-CGN promotes DC maturation and cytokine production through TLR4 signaling pathway **A.** On day 7, DCs were pretreated with or without TAK-242, and then treated with different doses of λ-CGN or LPS for 12 h. The levels of CD40 and CD86 were detected by flow cytometry (upper panels). Summary of CD40 and CD86 MFI is shown in lower panel. **B.** The supernatants of the above samples were collected to detect the secretions of IL-12 and TNF-α by ELISA. Data are from 3 independent experiments. *P* values were obtained by paired t-test. **C.** On day 7, DCs were treated with 100 μg/ml of λ-CGN. Cells were collected at the indicated time points and cytoplasmic proteins were isolated. The protein levels and phosphorylation status of molecules in TLR4 signaling pathway were detected by Western blot.

It has been shown that λ-CGN regulates immune responses through TLR4 signaling pathway [16, 17]. In order to detect the role of TLR4 signaling pathway in the maturation of DCs induced by λ-CGN, TLR4 inhibitor, TAK-242, was used to treat DCs before λ-CGN and LPS treatment. We observed that the pretreatment of TAK-242 significantly inhibited the expressions of CD40 and CD86 on DCs, and suppressed the secretions of IL-12 and TNF-α induced by λ-CGN and LPS (Figure 4A&4B). Moreover, the activation status of down-stream molecules in TLR4 signaling pathway was analyzed. After 100 μg/ml of λ-CGN treatment, DCs were collected at the indicated time points and cytoplasmic and nuclear proteins were extracted. The protein levels and phosphorylation status of the molecules in MAPK and NF-κB signaling pathways were detected by Western blot. After λ-CGN treatment, the phosphorylation of p38 and IκB was enhanced from 10 min and lasted to 240 min. The phosphorylation of ERK and NF-κBp65 was enhanced from 30 min and lasted to 240 min (Figure 4C). These results indicated that λ-CGN promoted DC maturation and cytokine production through TLR4 signaling pathway.

### HPV DC-based vaccine treated with λ-CGN inhibited tumor growth

**Figure 5:**
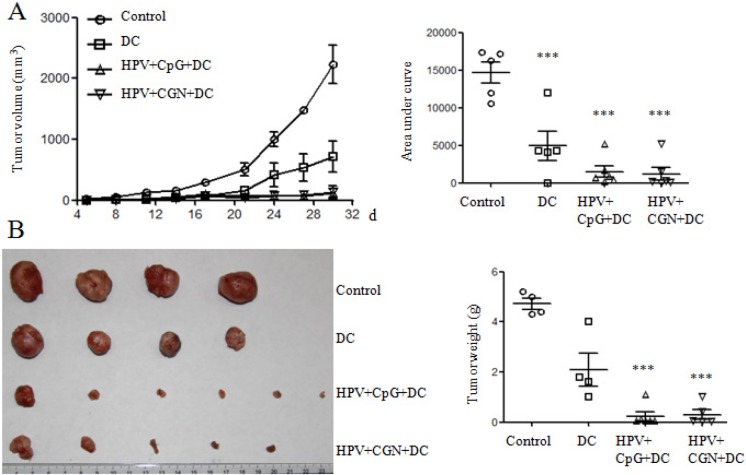
HPV DC-based vaccines suppressed tumor growth Tumor mice were immunized twice with DCs on days 3 and 10. **A.** The curves of tumor growth are shown in left panel. The area under curve (AUC) calculated using Prism 5 is shown in right panel. **B.** Tumors were isolated and weighted at the end of this experiment. ***P<0.001 (ANOVA) compared to control group.

The adjuvant effect of λ-CGN on HPV DC-based vaccine was detected in TC-1 tumor mouse model. After 3 days of the establishment of tumor mouse model, tumor mice were randomly divided into 4 groups (6 mice/group). Tumor mice were treated on days 3 and 10. Control group was received with PBS, DC group received λ-CGN treated DCs without HPV peptides, HPV+CpG+DC group received CpG treated DCs pulsed with HPV peptides (as positive control group) and HPV+CGN+DC group received λ-CGN treated DCs pulsed with HPV peptides. Compared with control group, HPV+CpG+DC and HPV+CGN+DC dramatically inhibited tumor growth. DC group also significantly inhibited tumor growth (Figure 5A). In control group, two tumor mice with tumor volumes 101 and 2385 mm^3^ died on days 11 and 29, respectively. One tumor mouse with tumor volume 21 mm^3^ died on day 17 and one mouse was tumor free in DC group. In HPV+CGN+DC group, one mouse was tumor free. Due to the large tumor volumes in control group, all tumor mice were sacrificed on day 30 to isolate tumors to take photo and weight. As shown in Figure 5B, HPV+CpG+DC and HPV+CGN+DC significantly decreased tumor weight compared to control group.

### The immune responses induced by HPV DC-based vaccine in tumor mice

**Figure 6:**
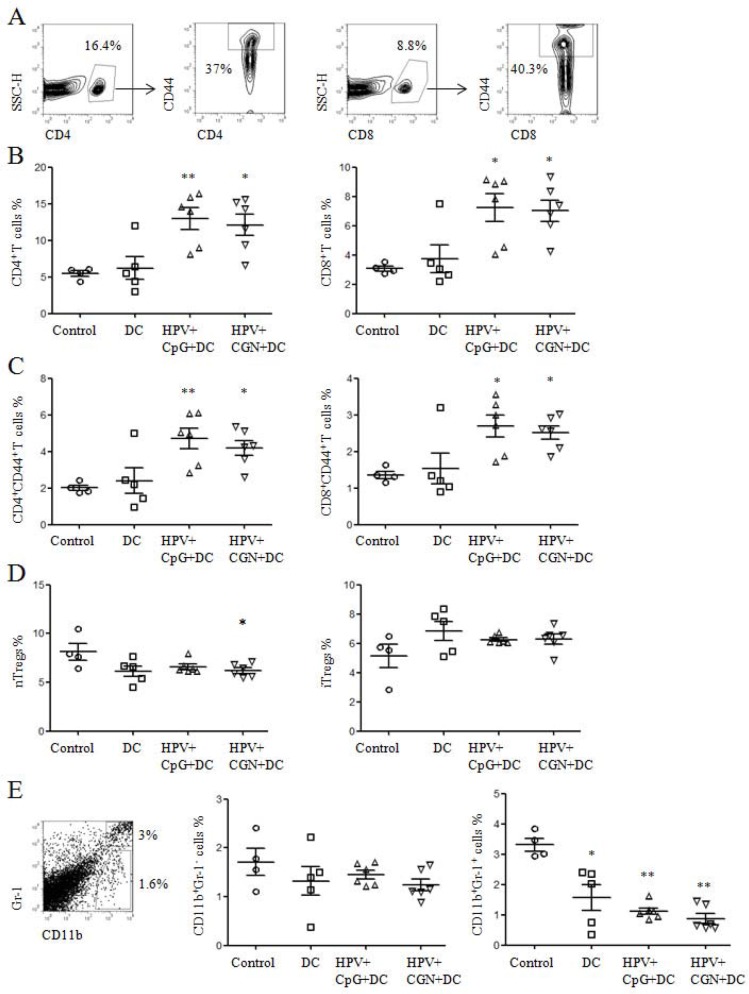
HPV DC-based vaccines activated CD4^+^ and CD8^+^ T cells and decreased nTregs and MDSCs **A.** At the end of this experiment, splenocytes were isolated from tumor mice to detect the frequencies of T cells and their activation status by flow cytometry. **B.** The frequencies of CD4^+^ and CD8^+^ T cells are shown. **C.** Summary of CD4^+^ and CD8^+^ T cell activation status is shown. **D.** The frequencies of nTregs and iTregs are shown. **E.** The frequencies of CD11b^+^Gr-1^−^ macrophages and CD11b^+^Gr-1^+^ MDSCs were detected by flow cytometry. Summary of macrophages and MDSCs are shown in right panels. *P<0.05 and **P<0.01 (ANOVA) compared to control group.

To investigate whether the antitumor effect is correlated with the immune responses induced by HPV DC-based vaccine, spleens were collected from the above tumor mice on day 30 to detect the frequencies of CD4^+^ and CD8^+^ T cells and their activation status, the frequencies of regulatory T cells (Tregs), macrophage and myeloid-derived suppressor cells (MDSCs), antigen-specific proliferation of splenocytes and CD8^+^ T cell responses. As shown in Figure 6A, CD4^+^ and CD8^+^ T cells were gated to analyze the frequencies of CD44^+^ T cells. We observed that the frequencies of CD4^+^ and CD8^+^ T cells in HPV+CpG+DC and HPV+CGN+DC groups were significantly higher than that of control group (Figure 6B). Moreover, the frequencies of CD4^+^CD44^+^ and CD8^+^CD44^+^ T cells were significantly increased in HPV+CpG+DC and HPV+CGN+DC groups (Figure 6C). Although DC group significantly inhibited tumor growth, the frequencies of CD4^+^ and CD8^+^ T cells and their activation status did not changed compared to the control group.

Tregs and MDSCs were involved in the immune suppression or dysfunction in tumor microenvironment [19–22]. We detected the frequencies of natural Tregs (nTregs: CD4^+^CD25^+^Foxp3^+^) and induced Tregs (iTregs: CD4^+^CD25^−^Foxp3^+^) in the above splenocytes. The frequencies of nTregs were significantly decreased in HPV+CGN+DC group compared with control group, but the frequencies of iTregs had no significant difference among these groups (Figure 6D). The frequencies of MDSCs and macrophages were also tested. Compared to control group, not only HPV+CpG+DC and HPV+CGN+DC groups but also DC group significantly decreased the frequencies of MDSCs (CD11b^+^Gr-1^+^). At the same time, all three treated groups did not change the frequencies of CD11b^+^Gr-1^−^ macrophages (Figure 6E).

**Figure 7:**
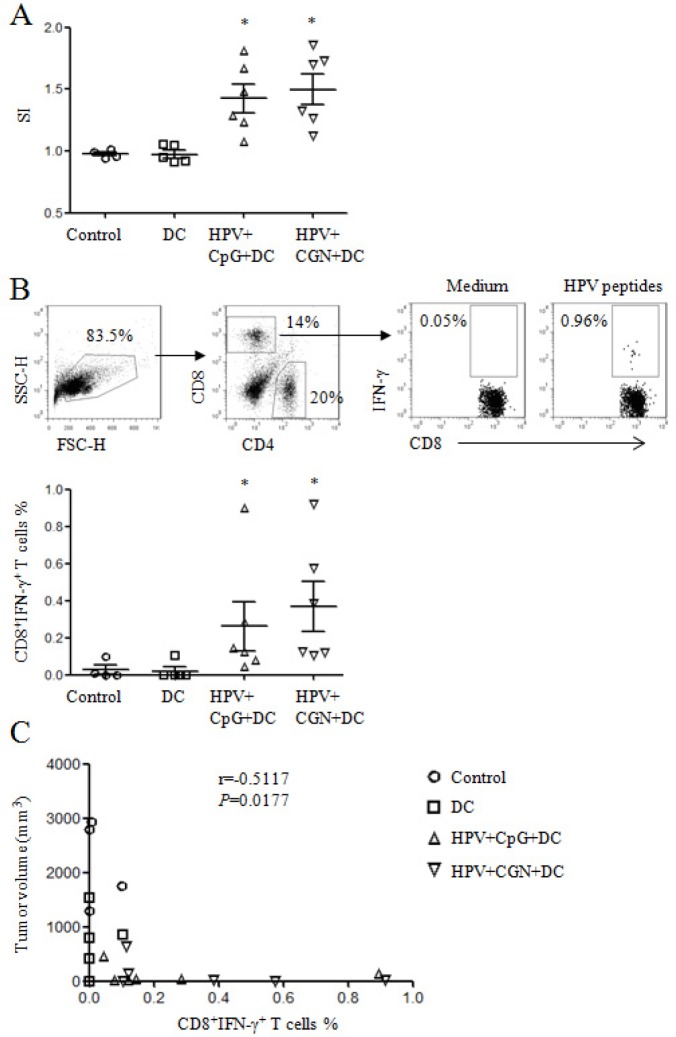
HPV DC-based vaccines induced antigen-specific cellular responses At the end of this experiment, splenocytes were isolated from tumor mice and stimulated with HPV-16 E6 and E7 peptides overnight to detect HPV-specific cellular responses. **A.** The proliferation of splenocytes was determined by MTT assay. The stimulatory index (SI) is shown. **B.** HPV-specific CD8^+^ T cell responses were analyzed by flow cytometry. Gating strategy is shown in upper panels and summary of HPV-specific CD8^+^ T cells is shown in lower panel. *p<0.05 (ANOVA) compared to control group. **C.** The correlation of tumor volumes and HPV-specific CD8^+^ T cell frequencies.

We further detected the antigen-specific cellular responses after HPV peptides stimulation. As shown in Figure 7A, HPV-specific proliferation was significantly increased in both HPV+CpG+DC and HPV+CGN+DC groups compared with control group. HPV+CpG+DC and HPV+CGN+DC also induced strong HPV-specific CD8^+^ T cell responses (Figure 7B), which is negatively correlated with tumor volume (on day 30) (Figure 7C). These results indicated that HPV-DC based vaccine suppressed tumor growth through the activation of T cells, inhibition of nTregs and MDSCs, and induction of HPV-specific cellular responses.

## DISCUSSION

In this study, we showed that the food additive, λ-CGN, promoted the maturation and cytokine production of DCs through TLR4 mediated signaling pathway. As adjuvant, λ-CGN treated HPV DC-based vaccine significantly suppressed tumor growth in TC-1 tumor mouse model, which might be correlated with the induction of HPV-specific CD8^+^ T cell responses, inhibition of nTregs and MDSCs, and activation of T cells.

It has been reported that CGN has the immunostimulating activities to increase macrophage phagocytosis, antibody production, lymphocyte proliferation, natural killer (NK) cell and NKT cell activity, proinflammatory cytokine secretion [14–16, 23, 24]. Here, we found that λ-CGN enhanced DC maturation, cytokine production and function, suggesting that the immunostimulating activities of CGN might be mediated by the activation of APCs. Moreover, the effect of λ-CGN on DC maturation and cytokine production is dependent on TLR4 signaling pathway, which is consistent with previous studies [16–18].

It has been shown that CGN can be used as adjuvant for peptide/protein to enhance immune responses and antitumor effect [12, 18]. We also detected the adjuvant effect of λ-CGN on DC-based vaccine. Compared to control group, HPV+CGN+DC significantly suppressed tumor growth. The antitumor effect of CGN treated HPV DC-based vaccine is similar with CpG treated HPV DC-based vaccine, a positive control, which has been used to enhance immune responses in animal models and clinical trials [25, 26]. Both CGN and CpG treated HPV DC vaccines promoted T cell activation, decreased the frequencies of MDSCs and induced antigen-specific cellular responses. All these factors might be contributed to the inhibition of tumor growth. Surprisingly, CGN treated DCs without HPV peptides also significantly inhibited tumor growth, which might be correlated with the decreased frequencies of MDSCs.

Due to the defective maturation and function of DCs in tumor microenvironment [27, 28], the antitumor efficacy of DC-based vaccine is dependent on not only the maturation of *ex vivo* cultured DCs but also the activation status of *in vivo* DCs. The combination of adjuvant and DC-based vaccine has been used to improve the antitumor effect [29]. Besides the adjuvant effect of CGN, it also reported that CGN has antitumor activities [12, 14, 15, 30, 31]. Therefore, in the future study, we will investigated the antitumor effect of co-administration of λ-CGN and HPV DC-based vaccine.

In conclusion, λ-CGN enhanced DC maturation and cytokine production through TLR4 signaling pathway. λ-CGN treated HPV DC-based vaccine induced strong antigen-specific cellular responses and suppressed tumor growth in tumor mouse model. These results suggested that λ-CGN might be a good candidate adjuvant for DC-based vaccine.

## MATERIALS AND METHODS

### Mice

C57BL/6 and BALB/c mice (6-8 weeks old) were obtained from Beijing laboratory animal research center (Beijing, China) and kept under pathogen-free conditions in a temperature-controlled, light-cycled animal facility of Xinjiang University. All animals were acclimatized for at least 1 week prior to experiments.

### Ethics statement

All methods were operated according to the guidelines approved by Xinjiang University. All animal experiments were approved by the Committee on the Ethics of Animal Experiments of Xinjiang Key Laboratory of Biological Resources and Genetic Engineering and carried out under the guidelines of the Animal Care and Use Committee of College of Life Science and Technology, Xinjiang University.

### The generation of bone marrow-derived DCs

Dendritic cells were generated from murine bone marrow cells in the presence of GM-CSF following the previous protocol [32]. On day 7, immature DCs were collected and treated with different concentrations (10, 50 and 100 μg/ml) of λ-CGN (Sigma-Aldrich). LPS (20 ng/ml) was used as positive control (Sigma-Aldrich). For TLR4 inhibitor experiments, immature DCs were pretreated with 1 μM TAK-242 (Medchem-express) for 1 h, and then treated with 0, 10, 50 and 100 μg/ml of λ-CGN or 20 ng/ml of LPS for 12 h. After treatment, supernatants and cells were collected to analyze cytokine secretion by enzyme-linked immunosorbent assay (ELISA) and DC maturation by flow cytometry, respectively.

For endocytosis analysis, λ-CGN treated DCs were incubated with the 50 μg/ml of FITC-Dextran (Sigma-Aldrish) at 37°C for 1 h. After washing with cold PBS, cells were stained with APC-CD11c (BD Biosciences) and analyzed by flow cytometry.

### Cytokine assay

The levels of IL-1β, IL-12 and TNF-α in supernatants of DC cultures were detected by ELISA kit according to the manufacturer's instructions (Elabscience, China). Absorbance at 450 nm was measured using an ELISA plate reader (Bio-Rad, USA). The concentrations of cytokines were calculated according to standard curve.

### Mixed lymphocyte reaction (MLR)

To examine the function of λ-CGN treated DCs, proliferation of splenocytes in MLR was estimated by MTT (Sigma-Aldrich) assay. Immature DCs were pretreated with 10 and 100 μg/ml of λ-CGN or 20 ng/ml of LPS for 12 h and then treated with 20 μg/ml mitomycin C for 30 min at 37°C. After washing with RPMI-1640, 50 μl of DCs were added to splenocytes (1×10^5^ cells/ml, 50 μl/well) at ratios of 1:5 and 1:10 in 96-well culture plate. After 2 days, supernatant was discarded and then 100 μl of MTT solution (0.5 mg/ml in RPMI-1640 medium) was added to each well. After 4 h, the formed formazan crystals were dissolved in 100 μl DMSO (Sigma-Aldrish). The OD540/655 values were measured by a 96-well microplate reader (Bio-Rad, USA).

### Western blots

Cell lysates were prepared by Nuclear and Cytoplasmic Extraction Kit (Beijing ComWin Biotech Co., Ltd) according to the manufacturer's instruction. Protein concentrations were detected by Pierce BCA Protein Assay Kit (Thermo, USA). Equal amounts of proteins were fractionated by 12% SDS-PAGE and transferred to nitrocellulose membranes. Membranes were blocked with 5% non-fat powdered milk (BBI life science, china) in TBS contained Tween 20 (TBST) for 1 h and then incubated with an appropriate dilution of primary antibody in TBST with 2% non-fat powdered milk for 2 h, followed by the incubation with HRP-conjugated secondary antibody for 1 h. Signals were detected using ECL assay kit (Beyotime Biotech Co., Ltd, China). All the antibodies purchased from Cell Signaling Technology.

### Tumor models and treatment

To determine the antitumor effect of HPV DC-based vaccine, C57BL/6 mice were subcutaneously implanted with TC-1 cells (1×10^5^ cells/mouse) on day 0, which constitutively expressed HPV-16 E6 and E7[33]. For preparation of HPV DC vaccine, bone marrow-derived DCs were treated with 100 μg/ml λ-CGN for 12 h. Then these λ-CGN treated DCs were pulsed with HPV-16 E6/E7 peptides including E6_43-57_ (QLLRREVYDFAFRDL), E6_53-62_ (AFRDLCIVYR), E7_11-20_ (YMLDLQPETT), E7_44-62_ (QAEPDRAHYNIVTFCCKCD) and E7_81-94_ (DLLMGTLGIVCPIC) (Shanghai Science Peptide Biological Technology Co., Ltd., China), and named as HPV+CGN+DC. 3 μg/ml CpG-ODN 1826 (InvivoGen) treated DCs pulsed with HPV-16 E6/E7 peptides (HPV+CpG+DC) were used as positive control. After 3 days, tumor mice were randomly divided into four groups (6 mice/group) and intradermally immunized with 50 μl PBS (control), 5×10^5^ CGN+DC, HPV+CpG+DC or HPV+CGN+DC in 50 μl PBS at peri-tumoral sites on days 3 and 10. The tumor volume was measured every two days and tumor volume was calculated as (long diameter) × (short diameter)^2^ × 0.5. When the average of tumor volumes reached 1500-2000mm^3^, mice were sacrificed and tumors were harvested and weighed. Splenocytes were collected to analyze immune responses. For analysis of antigen-specific proliferation, splenocytes were stimulated with HPV-16 E6/E7 peptides. After 48 h, proliferation was detected by MTT assay. Stimulatory index (SI) was calculated as OD_treated_/OD_untreated_.

### Flow cytometry

The viability of DCs was analyzed by Annexin V/PI staining kit (Shanghai Yeasen Biotechnology Co., Ltd., China) according to the manufacturer's instruction. For the phenotype analysis of DCs, λ-CGN treated cells were stained with fluorescence conjugated mAbs (PE-CD11c, APC-CD11c, APC-CD40, APC-CD80, FITC-CD86 and PE-MHC II) at room temperature for 15min.

To detect immune responses in spleen of tumor mice, the isolated splenocytes were used to analyze the frequencies of CD4^+^/CD8^+^ T cells, their activation status (CD44^+^), macrophages (CD11b^+^Gr-1^−^) Tregs (CD4^+^CD25^+/−^Foxp3^+^) and myeloid-derived suppressor cells (MDSCs, CD11b^+^Gr-1^+^). For analysis of HPV-specific cellular responses, splenocytes (1×10^6^/ml) were treated with HPV-16 E6/E7 peptides and cultured overnight in the presence of Golgi stop (BD Biosciences). Cell surface and intracellular staining was performed according to our previous study [34]. All the fluorescence conjugated mAbs were purchased from BD Biosciences. All samples were collected on FACSCalibur (BD Biosciences) and data were analyzed using FlowJo software (Tree Star, Inc., Ashland, OR).

### Statistical analysis

Statistical analysis was done by one-way analysis of variance (ANOVA) or paired t-test. *P* < 0.05 was considered to be statistically significant.

## Abbreviations

APCs: antigen presenting cells
CGN: carrageenan
CTL: cytotoxic T lymphocyte
DCs: dendritic cells
HPV: human papillomavirus
iTregs: induced regulatory T cells
MDSCs: myeloid-derived suppressor cells
MFI: mean fluorescence intensity
MLR: mixed lymphocyte reaction
nTregs: natural regulatory T cells
Th: helper T cell
TLR: toll-like receptor
